# Clinical benefits and risks of high-dose intravenous vitamin C: a systematic review

**DOI:** 10.25122/jml-2025-0176

**Published:** 2026-01

**Authors:** Abdulrahman Alangari, Jamal Arif, Fahd Al Qureshah, Fahad Alkhodairy

**Affiliations:** 1 Department of Research & Development, evercare, Riyadh, Saudi Arabia; 2 Department of Gastroenterology and Hepatology, Prince Sultan Military Medical City, Riyadh, Saudi Arabia; 3 College of Medicine, Shaqra University, Shaqra, Saudi Arabia; 4 Bioscience Program, King Abdullah University of Science and Technology (KAUST), Thuwal, Saudi Arabia

**Keywords:** Vitamin C, intravenous, high-dose, sepsis, oncology, home infusion, ARDS, acute respiratory distress syndrome, G6PD, glucose-6-phosphate dehydrogenase, IVC, intravenous vitamin C, MA, meta-analysis, PD-L1, programmed death ligand-1, RCT, randomized controlled trial, SR, systematic review, TSA, trial sequential analysis, STAT 1, signal transducer and activator of transcription 1

## Abstract

High-dose intravenous vitamin C (IVC) achieves plasma concentrations that are not attainable by oral administration and has been investigated as an adjunct in sepsis, oncology, and symptom management. To synthesize the evidence regarding the clinical benefits and risks of high-dose IVC, as well as the potential advantages of on-site infusion, a PRISMA-informed search of PubMed/PMC, Scopus, and Web of Science (2010–2025) was conducted, prioritizing randomized controlled trials, systematic reviews, and high-quality observational studies. Pharmacokinetic and mechanistic studies of IVC support plausible physiologic benefits through antioxidant effects, catecholamine biosynthesis, and immune modulation, with recent evidence showing down regulation of pro-inflammatory STAT1/PD-L1 pathways in experimental sepsis. Oncology phase I and II studies demonstrate safety and quality-of-life improvements; a randomized phase II pancreatic trial reported a promising survival benefit when combined with chemotherapy. Some of the major risks include oxalate nephropathy and hemolysis in patients with glucose-6-phosphate dehydrogenase deficiency, especially with very large or repeated doses, suggesting pre-screening to avoid these risks. Furthermore, the literature on home infusion and IV therapies is limited; however, the expanding home infusion infrastructure offers an avenue for monitored IVC delivery. In conclusion, evidence does not support routine use of high-dose IVC in sepsis, and its role in oncology remains supportive and exploratory, with potential risks requiring caution. Furthermore, interest in home-based infusion services is increasing in several healthcare systems, although clinical outcome data specific to high-dose IVC in these settings remain limited.

## INTRODUCTION

Vitamin C is an essential water-soluble micronutrient with roles as an enzymatic cofactor (collagen synthesis, catecholamine production), antioxidant actions, and modulation of immune responses [1]. Humans cannot synthesize vitamin C, and plasma concentrations are tightly regulated by intestinal absorption, renal reabsorption, and metabolic clearance. Oral vitamin C supplementation increases plasma concentrations only to a limited plateau due to saturable intestinal transporters. Intravenous administration bypasses these limits and can produce pharmacologic plasma concentrations (millimolar range) that have distinct biochemical effects compared with physiologic (micromolar) levels achieved orally [2].

One pharmacokinetic study demonstrated that IV vitamin C (IVC) dosing results in plasma concentrations 30–70 times higher than those achieved with the maximal tolerated oral dose [2]. This foundational insight stimulated clinical investigation of IVC in two broad directions: (i) correction of deficiency and supportive care (e.g., in critical illness, perioperative settings, or palliative oncology care), and (ii) pharmacologic, potentially cytotoxic uses in oncology and modulation of oxidative and inflammatory pathways in sepsis and acute respiratory failure [2,3].

Interest in high-dose IVC has accelerated over the last 15 years due to growing evidence of clinical benefits. Mechanistically, pharmacologic concentrations can generate hydrogen peroxide in the extracellular fluid, selectively increasing oxidative stress in tumor cells lacking robust catalase/peroxidase defenses, while normal cells are relatively protected, providing a biologic rationale for oncologic adjunct studies [3,4]. In sepsis and critical illness, proposed mechanisms include antioxidant scavenging of reactive oxygen species, restoration of endothelial barrier function, suppression of pro-inflammatory cascades, and enhancement of catecholamine biosynthesis through its role as a cofactor for dopamine β-hydroxylase, which could conceivably reduce organ dysfunction [5-8].

Recent mechanistic studies have provided additional insights into the immunomodulatory effects of vitamin C. Zhang *et al*. [7] demonstrated that high-dose vitamin C potentiates immune modulation in experimental sepsis through inhibition of signal transducer and activator of transcription 1 (STAT1) phosphorylation and negative regulation of programmed death ligand-1 (PD-L1), suggesting potential to alter sepsis immune phenotypes. These findings complement earlier observations of endothelial protection and microcirculatory improvements in preclinical models [8]. In addition, high-dose vitamin C synergizes with anti-PD-1 in a lymphoma mouse model [9] and in non-small cell lung cancer in in vitro and in vivo models [10].

Nevertheless, human trials yielded heterogeneous results. Landmark randomized studies in sepsis (e.g., CITRIS-ALI) reported mixed endpoints about interpretation (secondary endpoint signals vs primary endpoint negativity), while the large LOVIT trial raised some safety concerns with increased composite risk of death or persistent organ dysfunction in the treatment arm, highlighting the significance of dose/timing/patient selection and rigorous trial design [6,11]. Recent meta-analyses and systematic reviews have consistently emphasized heterogeneity across trials, with variable dosing regimens (ranging from ≈1500 mg/day to multi-gram protocols), timing, duration, and co-interventions complicating pooled interpretation [12-15]. However, a cautious approach with continuous monitoring of sepsis patients on high-dose IVC has been recommended [16].

In oncology, phase I and II trials over the past decade have generally shown that high-dose IVC is well tolerated when administered under monitoring, and several small randomized trials and observational studies suggest improvements in chemotherapy tolerance and patient-reported quality-of-life metrics. The recent randomized phase II PACMAN trial reported a notable survival difference when high-dose vitamin C was added to gemcitabine-based chemotherapy; although promising, these findings require confirmation in larger studies [3,17].

Safety concerns about vitamin C are nontrivial but pose a specific risk in certain exceptional clinical conditions. It is metabolized to oxalate, and prolonged or very high cumulative doses have been associated with oxalate nephropathy and dialysis requiring acute kidney injury in some case reports [1]. In addition, patients with glucose-6-phosphate dehydrogenase (G6PD) deficiency are at risk of hemolysis with very high doses. IVC can also interfere with point-of-care glucose measurements and interact with certain chemotherapeutic agents in complex ways. These specific risks suggest the necessity of risk stratification, pre-infusion testing, and safety monitoring [16,18-20].

The home-based infusion of IVC has rapidly developed its global market; however, in Saudi Arabia, formal data on clinical use of high-dose IVC remain sparse [21]. The national interest in expanding home-based infusion services and community nursing provides an infrastructure that could support monitored administration when clinically indicated. A narrative review of home infusion in Saudi Arabia described an expanding service landscape but emphasized the need for regulatory frameworks, training, and reporting critical elements if IVC is to be used safely in community settings [21].

This systematic review compiles updated clinical evidence for high-dose IVC across major indications (sepsis/critical illness, oncology, and supportive symptom control), details key safety findings, and provides recommendations for clinical adoption in home-based infusion.

## MATERIAL and METHODS

### Search strategy and information sources

A focused, PRISMA-informed literature search was conducted to identify human studies evaluating high-dose IVC, its clinical applications, safety profile, and delivery models, including home-based infusion services. Searches were performed in PubMed/MEDLINE, PMC, Scopus, and Web of Science, covering publications from January 2010 through October 2025. The search strategy combined controlled vocabulary and free-text terms related to the intervention and context, including “intravenous vitamin C,” “high-dose ascorbic acid,” “pharmacological ascorbate,” “IV ascorbate sepsis,” “IV vitamin C cancer,” “oxalate nephropathy vitamin C,” and “home infusion Saudi Arabia.” Reference lists of relevant reviews and included articles were manually screened to identify additional eligible studies.

### Eligibility criteria

#### Inclusion criteria

Studies were included if they met the following criteria:

*Population:* Human participants of any age receiving high-dose IVC in clinical, outpatient, or home-based settings.

*Intervention:* IVC at pharmacologic or high-dose regimens, including use as adjunctive therapy.

*Comparator:* Placebo, standard care, oral vitamin C, inpatient infusion, or no comparator. Studies without a comparator were included if they provided relevant safety or implementation data.

*Outcomes:* Clinical efficacy outcomes, safety and adverse events, and implementation or service-delivery outcomes relevant to hospital or home infusion contexts.

*Study design:* Randomized controlled trials, systematic reviews and meta-analyses, observational studies, and case reports describing safety signals or service implementation.

*Publication characteristics:* Peer-reviewed publications in English, published between January 2010 and October 2025.

#### Exclusion criteria

Studies were excluded if they involved only animal models, did not include IVC administration, lacked extractable outcome data, reported duplicate datasets, or were not available in English. However, preclinical and animal studies were cited selectively to support the mechanistic rationale where relevant.

### Study selection process

Two reviewers (JA and FAK) independently screened titles and abstracts using predefined eligibility criteria. Disagreements were resolved through discussion and consensus. Full-text articles were retrieved for all potentially eligible studies and independently assessed by the same reviewers. Studies were excluded at this stage if they did not meet the inclusion criteria or contained overlapping or redundant data.

### Data extraction

Data extraction was performed independently by two reviewers (JA and FAK) using a standardized approach. Extracted information included study design, population characteristics, dosing regimens and schedules, concomitant therapies, primary and secondary outcomes, adverse events, and key methodological limitations. Discrepancies were resolved through discussion.

### Data compilation and analysis

Given substantial clinical and methodological heterogeneity across included studies, including variations in dosing, timing, duration, patient populations, and outcome measures, a narrative synthesis was undertaken. Studies were grouped by clinical indication and infusion setting, with particular attention to safety signals and implementation considerations. Formal meta-analysis was not performed due to heterogeneity. Further, a formal risk-of-bias tool was also not applied; instead, methodological quality was appraised narratively based on study design, sample size, internal validity, and consistency of findings.

## RESULTS

**Figure 1:**
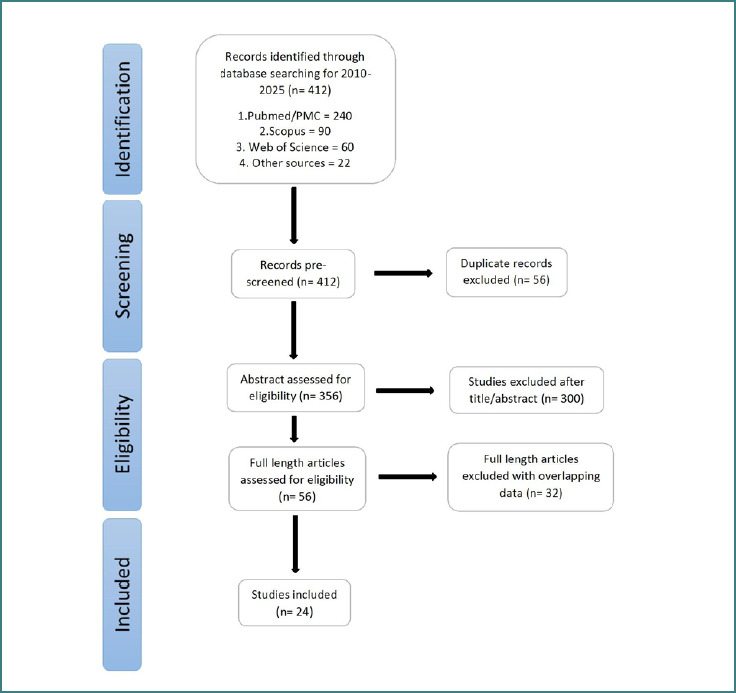
Preferred Reporting Items for Systematic Reviews and Meta-analysis (PRISMA) flow diagram for selection of relevant studies

A total of 412 publications were identified across all the searched databases. After removing 56 duplicate records, 356 were screened for eligibility. Of these, 300 studies were excluded after initial screening of the title or abstract. A total of 56 full-length articles were assessed for eligibility, and a further 32 articles were excluded due to overlapping data. Only 24 studies were included in the final analysis. A PRISMA flow chart depicting the study selection process is shown in Figure 1.

We present results by clinical indication on pharmacokinetics, sepsis/critical illness, oncology, other clinical contexts, and safety signals. It is further stated that the review represents a systematic review with narrative synthesis rather than a quantitative meta-analysis, due to substantial clinical and methodological heterogeneity across included studies. In this review, ‘high-dose’ IVC is operationally defined as regimens delivering pharmacologic plasma concentrations, typically ≥10,000 mg per infusion or ≥50 mg/kg/day, acknowledging that dosing was variably reported as weight-based or absolute doses across studies. A summary of selected randomized and high-quality trials is provided in Table 1.

**Table 1:** Selected human trials of high-dose IVC, primary outcome, and key results

Study (Year)	Design	Population	Dose/Schedule	Primary outcome indicator	Key result/comment
Fowler *et al*. (2019) [5]	RCT (CITRIS-ALI)	Severe sepsis/ ARDS (*n* = 167)	50 mg/kg IV every 6h x96h (~15,000 mg/day)	Organ dysfunction, biomarkers	Primary biomarker endpoints were negative; secondary analysis suggested reduced mortality
Lamontagne *et al*. (2022) [6]	RCT (LOVIT)	Adults with sepsis on vasopressors (*n* = 872)	50 mg/kg IV every 6h x4 days	Death or persistent organ dysfunction at 28d	Higher risk in the vitamin C treatment arm; no benefit
Lee *et al*. (2023) [12]	SR & MA with TSA	IVC monotherapy RCTs	Various	Mortality	Found heterogeneous trial results; uncertainty
Wen *et al*. (2023) [13]	SR & MA	Sepsis RCTs pooled (24 trials)	Various	Mortality, organ dysfunction	Borderline short-term mortality signal but substantial heterogeneity; not definitive
Zeng *et al*. (2023) [14]	SR & MA	High-dose IVC trials	Various	Clinical outcomes	Mixed conclusions across high-dose trials; benefit not consistent
Bodeker *et al*. (2024) [3]	Randomized Phase II (PACMAN)	Metastatic pancreatic cancer (*n* = 34)	75,000 mg IV 3x/ week + chemo	Overall survival	Reported doubled median overall survival (8 to 16 months); small sample, needs confirmation
Mussa *et al*. (2022) [23]	Narrative/ systematic	Oncology trials (phase I-II)	50,000-100,000 mg infusions 2-3x/week	Safety, quality of life, tumor response	Generally safe; quality of life improved; efficacy signals variable
Fontana *et al*. (2020) [18]	Case series	AKI with oxalate nephropathy	Variable; excessive cumulative doses	Renal biopsy-proven oxalate nephropathy	Highlighted renal risk with high cumulative doses
Wang *et al*. (2024) [19]	Case reports & review	G6PD deficiency cases	High-dose IVC	Hemolytic anemia	All reported G6PD patients developed hemolysis; caution is advised

ARDS, acute respiratory distress syndrome; G6PD, glucose-6-phosphate dehydrogenase; MA, meta-analysis; RCT, randomized controlled trial; SR, systematic review; TSA, trial sequential analysis

### Pharmacokinetics and mechanistic rationale of high-dose IVC

Intravenous administration achieved plasma vitamin C concentrations orders of magnitude greater than oral dosing, enabling prooxidant chemistry in extracellular fluid such as hydrogen peroxide generation, and achieving concentrations that may interact with tumor microenvironments or modulate oxidative injury in sepsis [2,5,22,23]. These pharmacokinetic features form a consistent biological rationale across indications but require rigorous testing.

Recent mechanistic work has elucidated additional pathways. Vitamin C supports redox balance and may reduce endothelial injury and microcirculatory dysfunction in preclinical models and ex vivo assays [8]. As a cofactor for dopamine β-hydroxylase, vitamin C may enhance norepinephrine synthesis; randomized physiologic data showing improved norepinephrine endpoints are consistent with this mechanism [24].

### High-dose IVC in sepsis and critical illness

Sepsis has been the most studied critical-care indication. The CITRIS-ALI randomized trial used 50 mg/kg every 6h for 96 hours and found no improvement in primary biomarker endpoints but observed a reduction in mortality in a secondary analysis, prompting debate about study power and multiplicity [5]. The larger LOVIT trial randomized 872 adults receiving vasopressors to IVC or placebo and reported a higher composite risk of death or persistent organ dysfunction at 28 days in the vitamin C group, leading to caution about routine IVC in sepsis [6].

Smaller trials and observational studies have reported variable potential benefits; the negative composite outcome observed in the large LOVIT trial carries greater evidentiary weight. A few single-center RCTs showed improvements in vasopressor requirements or biomarker endpoints, while others found no clear benefit [24,25]. A retrospective study suggested improved 28-day survival in patients with sepsis-associated acute kidney injury who received vitamin C, though observational data require cautious interpretation [26]. These findings underscore that current evidence does not support routine use of high-dose IVC in sepsis. Further, recent multiple meta-analyses suggested that trial heterogeneity (dose, timing, co-interventions, populations) and potential biases limit definitive conclusions and that larger harmonized trials are necessary for routine clinical adoption of IVC [12-15].

In addition, high-dose vitamin C has been shown to downregulate pro-inflammatory signaling in experimental sepsis by inhibiting STAT1 phosphorylation and negatively regulating PD-L1 in cecal ligation and puncture models, suggesting potential to alter sepsis immune phenotypes [7].

### High-dose IVC in oncology

Phase I-II oncology trials have generally shown tolerability of high-dose IVC (50,000–100,000 mg per infusion 2–3 times weekly) and reported improved chemotherapy tolerability and patient-reported quality of life measures [27]. The randomized phase II PACMAN trial observed a longer median overall survival in metastatic pancreatic cancer when high-dose IVC was added to gemcitabine/nab-paclitaxel; however, this finding was derived from a small sample size and remains exploratory [3,17].

A comprehensive review by Mussa *et al*. [23] observed in oncology trials that high-dose vitamin C is generally safe when administered under monitoring, with improvements in quality-of-life metrics and chemotherapy tolerance. However, efficacy signals remain variable and require confirmation in larger studies.

Mechanistic studies continue to support the prooxidant hypothesis that the pharmacologic concentrations of vitamin C generate hydrogen peroxide in the extracellular space, which may selectively increase oxidative stress in tumor cells lacking robust catalase/peroxidase defenses [3,4]. This biological rationale, combined with promising early clinical signals, justifies continued investigation in well-designed oncology trials.

Table 2 presents a summary of expert-opinion evidence informed by published trials and systematic reviews, rather than a formal GRADE assessment.

**Table 2:** Quality of evidence and recommendations for key indications

Indication	Quality of evidence	Effect estimate/summary	Recommendation
Sepsis/critical illness	Low to Moderate (inconsistent RCTs)	Heterogeneous; trend to reduced short-term mortality in pooled analyses, but LOVIT negative and recent metaanalyses show no consistent benefit [12-15]	Not routine; use only in trials or selected contexts with monitoring [16]
Oncology (adjunctive)	Low (phase I-II data; small RCTs)	Tolerable; quality of life benefits; limited survival signal (small trials) [3,23]	Consider for supportive care
Supportive symptom control (fatigue, recovery)	Very low	Anecdotal/small uncontrolled studies	Can be used with monitoring
Safety (renal, hemolysis)	Moderate (case reports + observational)	Oxalate nephropathy and G6PD hemolysis documented [18,19]	Pre-infusion renal testing and G6PD caution; registry reporting advised [16]

### Other clinical contexts

IVC was widely tested during the COVID-19 pandemic, with mixed results. It generally did not show a consistent mortality benefit in heterogeneous ICU populations [28]. For symptomatic interventions (fatigue, post-viral recovery), evidence remains limited to small studies or observational reports.

An interesting application has been explored in allogeneic hematopoietic cell transplant recipients, where IVC supplementation has shown a promising impact on clinical outcomes in preliminary studies, although larger trials are needed [27,29].

### Miscellaneous reports on high-dose IVC

**Oxalate nephropathy:** Multiple case reports described biopsy-proven oxalate nephropathy after repeated or excessive IVC, sometimes leading to dialysis dependence. These reports recommend caution, especially in patients with reduced renal reserve [18,30]. A review by Rosenstock *et al*. comprehensively discussed risk factors for oxalate nephropathy and monitoring strategies [30]. In addition, Honore *et al*. emphasized the need to monitor safety and oxalate levels when dosing vitamin C in critically ill patients [20].

**Hemolysis in G6PD deficiency:** A few studies also indicated that patients with G6PD deficiency exposed to high-dose IVC developed hemolytic anemia [19,31]. A screening or avoidance in at-risk populations is prudent [19,31].

**Glucose measurement interference:** High-dose IVC can cause interference with certain point-of-care glucose meters, producing spuriously high readings, which is an operational safety consideration in ICU glycemic management [16].

**Abrupt cessation hypothesis:** Hemilä and Chalker [32] performed a secondary analysis of the LOVIT trial and proposed that abrupt termination of vitamin C after short IV courses could plausibly explain adverse signals, though this remains exploratory [32].

**Other concerns:** Infusion site reactions, fluid overload, and theoretical interactions with chemotherapeutic agents should be considered and discussed with treating teams [16,23].

### Clinical practice recommendations

Published data specifically evaluating high-dose IVC in Saudi Arabia are scarce. However, Saudi healthcare has documented growth in home-based infusion services and community-based care models. The expanding home infusion infrastructure presents both opportunities and challenges. While it could improve access to supportive therapies for patients with advanced cancer or chronic conditions, it requires robust regulatory oversight to prevent inappropriate use and ensure patient safety [21].

Based on the current evidence synthesis, we propose the following prioritized actions:

1. **Patient selection criteria** based on evidence-based indications.
2. **Baseline testing**, including renal function and G6PD screening, is particularly relevant in Middle Eastern populations, given the higher G6PD deficiency prevalence.
3. **Infusion monitoring** with trained personnel and emergency response capability.
4. **Adverse event registries** to establish local incidence and risk factors.
5. **Quality assurance** frameworks aligned with international best practices.

Collaboration between oncology, critical care, and nephrology services will be necessary to ensure safety.

**Figure 2:**
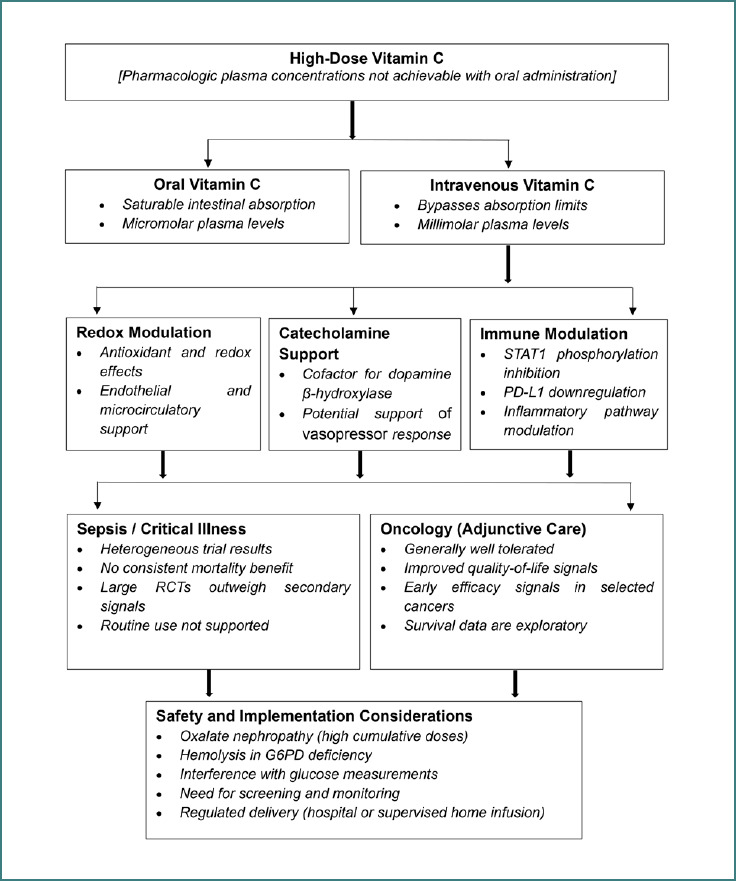
Schematic overview of pharmacokinetics, mechanistic pathways, clinical contexts, and safety considerations of high-dose IVC

Figure 2 illustrates pharmacokinetic differences between oral and intravenous administration of vitamin C, proposed mechanistic pathways (redox modulation, catecholamine biosynthesis, and immune regulation), major clinical contexts (sepsis/critical illness and oncology supportive care), and key safety and implementation considerations.

## DISCUSSION

This review brings together current clinical and experimental evidence on high-dose IVC and highlights both its potential and its limitations. A clear pharmacokinetic rationale supports high-dose IVC, as intravenous administration achieves plasma concentrations not attainable via oral administration. Within this framework, the existing literature suggests promising signals in selected clinical settings, particularly in oncology supportive care and early-phase cancer trials [17,23,27]. In these contexts, IVC has been associated with acceptable tolerability and improvements in patient-reported outcomes, with some preliminary indications of efficacy when used as an adjunct to standard anticancer therapies [17, 23, 27].

Mechanistic studies have strengthened the biological plausibility of these observations. High-dose IVC has been shown to exert antioxidant and redox-modulating effects, support catecholamine biosynthesis by serving as a cofactor in dopamine β-hydroxylase activity, and influence immune regulation. More recently, immune-modulatory effects, including inhibition of STAT1 phosphorylation and downregulation of PD-L1 expression, have been described, providing insight into how IVC may modify inflammatory and immune responses in both sepsis and cancer models [7,8,24]. These mechanistic findings offer a coherent explanation for observed clinical effects but do not, on their own, establish clinical benefit.

In contrast, evidence from sepsis and critical illness remains inconsistent. Despite extensive investigation, multiple contemporary meta-analyses published between 2023 and 2024 report substantial heterogeneity across trials, with no reproducible mortality benefit attributable to high-dose IVC [12–15]. Differences in patient populations, illness severity, timing of administration, dosing intensity, duration of therapy, and concomitant treatments complicate interpretation and limit the generalizability of findings. Collectively, these data do not support the routine use of IVC in sepsis at present and underscore the importance of refined trial design and patient selection.

Safety considerations remain central to the evaluation of high-dose IVC. Although serious adverse effects are uncommon, documented risks including oxalate nephropathy, hemolysis in individuals with G6PD deficiency, and interference with point-of-care glucose measurements necessitate structured risk mitigation strategies [16,18–20,30]. These risks highlight the need for baseline renal assessment, G6PD screening where appropriate, and careful monitoring during administration. The wide variation in dosing regimens and treatment protocols across published studies further complicates safety assessment and reinforces the need for standardized approaches [12,13].

From a healthcare system perspective, particularly in Saudi Arabia and similar settings, the rapid expansion of home-based infusion services presents both opportunities and challenges. When clinically indicated, such infrastructure could enable monitored delivery of IVC outside hospital settings; however, this requires clearly defined regulatory oversight, trained personnel, standardized protocols, and robust adverse-event reporting systems to ensure patient safety and appropriate use [21].

### Study Limitations

Several limitations should be considered when interpreting this review. The published literature exhibits wide variability in study populations, dosing regimens, treatment durations, and outcome measures, which restricts direct comparison and weakens the pooled conclusions. Many oncology studies are small or exploratory, limiting the strength of efficacy claims. Additionally, much of the safety evidence relies on case reports and retrospective data, which may not accurately capture the true incidence of adverse events. Finally, region-specific data on clinical implementation, particularly outside hospital settings, remain scarce.

### Future perspectives and development

Future progress in high-dose IVC research will depend on well-designed, adequately powered clinical trials using harmonized dosing strategies, standardized outcomes, and biologically informed patient selection. Integration of mechanistic biomarkers and prospective safety registries will be essential to better define benefit–risk profiles and to capture uncommon but serious adverse events. Within Saudi Arabia, the Vision 2030 agenda prioritizes modernization of healthcare delivery, including expansion of community-based and home healthcare services. This evolving infrastructure could support the monitored administration of IVC when clinically indicated, provided its use is governed by clear regulatory oversight, standardized protocols, trained personnel, and robust safety reporting systems. Aligning future research with healthcare policy and service development will be critical to establishing the appropriate role of high-dose IVC in clinical practice.

## CONCLUSION

High-dose IVC produces pharmacologic effects that are not achievable with oral supplementation and has demonstrated acceptable safety under controlled conditions. While supportive benefits are most evident in selected oncology settings, evidence in sepsis and critical illness remains inconsistent and does not support routine use. Given the presence of identifiable risks, clinical application should be cautious, selective, and supported by appropriate screening and monitoring. Continued high-quality research is necessary to clarify where, and for whom, high-dose IVC may offer meaningful clinical value.
